# Developing a set of ancestry-sensitive DNA markers reflecting continental origins of humans

**DOI:** 10.1186/1471-2156-10-69

**Published:** 2009-10-27

**Authors:** Paula Kersbergen, Kate van Duijn, Ate D Kloosterman, Johan T den Dunnen, Manfred Kayser, Peter de Knijff

**Affiliations:** 1 Department of Human Biological Traces (R&D), Netherlands Forensic Institute, PO Box 24044, 2490 AA The Hague, The Netherlands; 2 Department of Human and Clinical Genetics, Leiden University Medical Center, PO Box 9600, 2300 RC Leiden, The Netherlands; 3 Department of Forensic Molecular Biology, Erasmus University Medical Center, PO Box 2040, 3000 CA Rotterdam, The Netherlands

## Abstract

**Background:**

The identification and use of Ancestry-Sensitive Markers (ASMs), i.e. genetic polymorphisms facilitating the genetic reconstruction of geographical origins of individuals, is far from straightforward.

**Results:**

Here we describe the ascertainment and application of five different sets of 47 single nucleotide polymorphisms (SNPs) allowing the inference of major human groups of different continental origin. For this, we first used 74 cell lines, representing human males from six different geographical areas and screened them with the Affymetrix Mapping 10K assay. In addition to using summary statistics estimating the genetic diversity among multiple groups of individuals defined by geography or language, we also used the program STRUCTURE to detect genetically distinct subgroups. Subsequently, we used a *pairwise *F_ST _ranking procedure among all pairs of genetic subgroups in order to identify a single best performing set of ASMs. Our initial results were independently confirmed by genotyping this set of ASMs in 22 individuals from Somalia, Afghanistan and Sudan and in 919 samples from the CEPH Human Genome Diversity Panel (HGDP-CEPH)

**Conclusion:**

By means of our *pairwise *population F_ST _ranking approach we identified a set of 47 SNPs that could serve as a panel of ASMs at a continental level.

## Background

Forensic DNA profiling of biological crime scene samples of human origin is typically performed for identification of individuals, by matching the DNA profiles obtained to victims, suspects, or occasionally, missing persons. When a multilocus DNA profile from a crime scene sample fully matches the DNA profile of, say, a suspect, forensic laboratories often report a random match probability, commonly referred to as "match probability". The match probability reflects the probability of sampling a random individual with an identical multilocus genotype as found in the crime-scene stain (and matching the suspect) and is computed for different populations using population specific allele frequencies for which *a priori *assumptions about the genetic ancestry of the crime scene sample "donor" have to be made [1,2]. Inferring the geographic origin of the (unknown) donor can give an extra dimension to criminal investigation, e.g. narrowing down or diverting the DNA dragnet for the police [1-3]. However, ascertainment of the genetic ancestry of sample donors can only be done reliably if two basic requirements are met: (i) the availability of a suitable set of ancestry-sensitive markers (ASMs), and (ii) a reference database with global frequencies of such ASMs. These two requirements and the potential importance of the ability to be able to identify the geographical origin of unknown suspects of crimes was recognized by Dutch politicians, and in 2003 the Dutch parliament passed a law providing the legal frame work that could not only facilitate research on ASMs but also provided the legal possibility to actually apply them in forensic case work [4].

Loci exhibiting significant allele frequency differences among groups of individuals stratified on the basis of geography are often called ancestry-informative markers (AIMs). However, as indicated above, we prefer to use the term ancestry-sensitive markers (ASMs) because in our opinion *ancestry sensitivity *better reflects the uncertainties related to such marker whereas *ancestry informativity *implies that they clearly reveal ancestry. ASMs are already utilised for genetic association studies, drug response testing, admixture mapping and reconstructing evolutionary histories [5-9]. In a strict sense, the first ASMs to be developed and used for forensic applications were mitochondrial DNA (mtDNA) and Y-chromosomal polymorphisms [10-12]. An obvious disadvantage of the use of mtDNA and Y-chromosomal polymorphisms is the fact that they only provide information concerning the strict maternal or paternal ancestry. Therefore, they can only detect genetic admixture, when contrasting information of both marker types is obtained, but such admixture cannot be quantified based on such markers. By means of mtDNA and Y-chromosomal polymorphisms it is also extremely difficult to differentiate recent from ancient admixture episodes in the history of an individual. Especially in a forensic application, where ancient admixture events are rarely relevant but where recent admixture events can leave misleading mtDNA and Y-chromosomal signatures, this is a clear disadvantage. In order to obtain a more accurate inference of an individual's genetic ancestry including the identification of possible recent genetic admixture, autosomal DNA markers are more suitable.

Different sets of autosomal ASMs with at least a continental resolution have already been identified and published [5,10,13-15], but the available marker panels are still far from perfect, mainly due to different ascertainment strategies and reference datasets. Different approaches have been suggested to ascertain ASMs, including (i) BLAST searches comparing sequence variation among individuals from different populations [3], (ii) selecting SNPs in databases by focussing on extreme allele frequency differences values between populations [16], and (iii) exploring existing SNP panels with tens to hundreds of genetic markers via commercially available SNP-genotyping arrays [3,17-19]. Many of these previous studies first used non-genetic criteria such as geography or language in order to *a priori *stratify the study populations. Especially in the case of geographically distinct populations that could possibly share a similar genetic make-up, this is not always the most optimal strategy.

Here, we apply STRUCTURE, a software package that is widely used for detecting subgroups of genetically similar individuals among large populations [16-21], to identify suitable sets of ASMs, using the single nucleotide polymorphism (SNP) genotyping results obtained by means of the Affymetrix GeneChip^® ^Human Mapping 10K Array Xba131 (Mapping 10K array) [22-24] in the Y Chromosome Consortium (YCC) cell line panel [25]. With this panel we compared five different sets of ASMs that were identified on the basis of two statistical estimates summarizing overall genetic diversity among geographically predefined populations, F_ST _[[26], see also the methods section] and the Informativeness of ancestry Index (*I*_n_) [7], and two ascertainment procedures based on *pairwise *F_ST _comparisons. This resulted in the selection of a set of SNPs that could be used as forensically relevant ASMs. Subsequently, these ASMs were further tested in two independent sets of samples: (i) individuals from Somalia, Afghanistan, and Sudan, and (ii) the CEPH Human Genome Diversity Panel (HGDP-CEPH) [27,28].

## Results

### ASM ascertainment from 10K SNP array data

**Figure 1:**
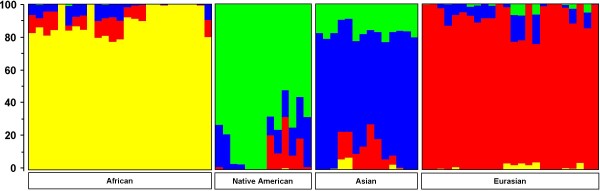
**STRUCTURE analyses of 8,474 SNPs among 74 globally dispersed individuals**. A STRUCTURE analysis revealed that the likelihood of the data was maximal at K = 4 ancestral populations (also called subgroups or clusters). Individuals are represented as vertical bars that are partitioned into segments corresponding to their membership of the clusters indicated by the four different colours. Each colour reflects the estimated relative contribution of one of the four clusters to that individual's genome and sum up to 100% (indicated at the Y-axis). Individuals were sorted according to their geographical origins (indicated below each group) after completion of the STRUCTURE analyses. E.g. for the left-most individual, sampled in Africa, there is about 82% relative contribution of the ancestral population represented by yellow, about 6% is attributed to the blue ancestral population and the remaining 12% is attributed to the red ancestral population. From this figure it becomes clear that this could be interpreted as a contribution of 82% - 12% - 6% of African, European, and Asian "genes" to the genome of this African individual. The results in this figure are based on the 8,474 SNPs genotyped in the 74 YCC cell lines from individuals from Africa (n = 25), Native American origin (n = 12), Asia (n = 14), and Eurasia (n = 23).

By means of STRUCTURE analyses we screened the 74 worldwide YCC samples from six human populations that were defined by their distinct geographical locations (referred to as 6geo) for the presence of genetically defined subgroups on the basis of the total set of 8,474 SNPs per individual after quality control (Figure 1). The six geographical locations with defined human populations (6geo) are (i) (Central) Africa, (ii) South Africa, (iii) Asia (including Pakistan), (iv) Europe, (v) Northern Asia (Russia and Siberia), and (vi) Native America. All STRUCTURE models indicated a best fit of the data at four clusters (K = 4). These four clusters combined the six geographically (6geo) defined populations into the following four genetic subgroups (referred to as 4gen): (i) African, composed of individuals from South Africa and Central Africa, (ii) Native American, composed of individuals of Native American origins from North, Central and South America, (iii) Asian, including individuals from East Asia and Siberia, and (iv) Eurasian, including individuals from Russia, Europe, and Pakistan. These results also suggested that the identification of suitable ASMs could be best achieved by comparing the *posteriori *defined four genetic subgroups (4gen) and not - as is often done - by comparing the *a priori *geographically defined populations (in our case six, hence 6geo). For the purpose of comparison we describe below the use of ASMs identified by means of both strategies (4gen and 6geo).

**Figure 2:**
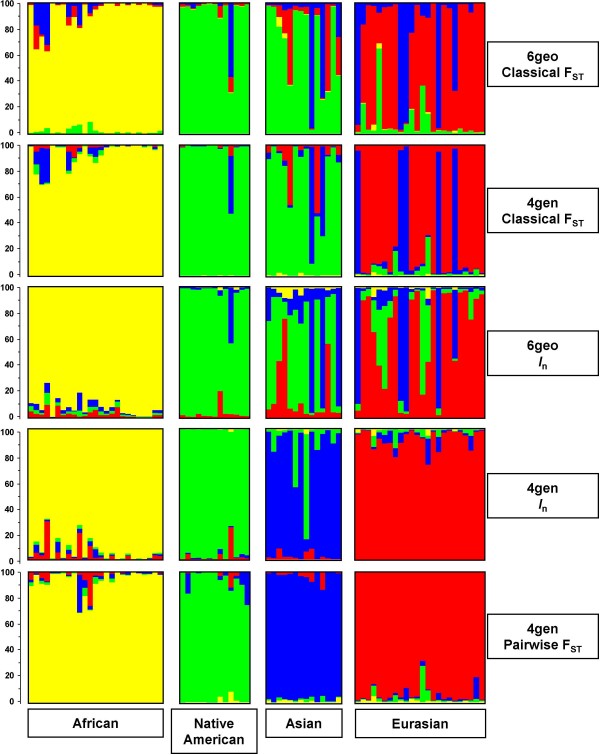
**Different approaches for the ascertainment of ASMs**. The five panels show the results from STRUCTURE analyses for five different sets of 47 ancestry sensitive markers (ASMs) among 74 globally dispersed individuals from Africa (n = 25), Native American origin (n = 12), Asia (n = 14), and Eurasia (n = 23). To the right of each panel we indicate which statistical approach is used to identify each set of ASMs (see methods for more details). For each set of 47 ASMs, the likelihood of the data was maximal at K = 4 ancestral populations (clusters). Individuals are represented as vertical bars that are partitioned into to segments corresponding to their membership of the clusters indicated by the four different colours. Each colour reflects the estimated relative contribution of one of the four subgroups to that individual's genome and sum up to 100% (indicated at the Y-axis). Individuals were sorted according to their geographical origins (indicated below each group) after completion of the STRUCTURE analyses.

Since one of the aims of our study was to explore and compare different methods and selection criteria one could use to identify the most optimal set of ASMs from exactly the same set of populations we used five different methods that resulted in five different sets of 47 ASMs. These five sets, identified via *classical *and *pairwise *F_ST _calculations, (see methods for further explanation), as well as based on *I*_n_, were: (i) 6geo *classical *F_ST_, (ii) 4gen *classical *F_ST_, (iii) 6geo *I*_n_, (iv) 4gen *I*_n_, and (v) 4gen *pairwise *F_ST _(Figure 2). Marker overlap among these sets consisted of only five African-specific SNPs. The remaining ASMs in three sets (i, ii and iii) out of five were also mainly African-specific (not shown). This suggested a bias towards the African population in ASM ascertainment by these identification strategies, which is not unexpected given the often observed substantial genetic differentiation between African and non-African groups. This was also confirmed by a retrospective F_ST _analysis of all 74 YCC samples grouped into the four genetically defined subgroups or six geographically defined subgroups with each of the five different sets of 47 ASMs (see additional file 1: F_ST _analyses of the five different sets of ASMs among the 74 YCC samples). Only the 4gen *pairwise *F_ST _selected set of 47 ASMs displayed a relative uniform *pairwise *F_ST _distribution among all population pairs, whereas the other marker sets, but especially the two selected using *I*_n _showed markedly elevated F_ST _values among all population pairs contrasting African vs. non-African populations and substantially lower *pairwise *F_ST _values among all non-African pairs of populations. STRUCTURE analyses were performed on the data of the YCC panel for each of the five sets of 47 ASMs (Figure 2). The 6geo *classical *F_ST _and the 4gen *classical *F_ST _clustering revealed an almost identical subgrouping of the YCC data (Figure 2), with three genetically distinct subgroups (African, Eurasian, and the combined Asian/Native American group). The combined Asian/Native American groups detected here contrasts with the clustering using the total number of 8,474 SNPs where both groups were identified separately (Figure 1). Also the 6geo *I*_n _clustering revealed a similar three subgroup structure (Figure 2), whereas the 4gen *I*_n _clustering showed four distinct subgroups as the analyses using all 8,474 SNPs did (African, Asian, Native American, Eurasian, see Figure 1). The same four-group clustering was also obtained based on the 4gen *pairwise *F_ST _approach, but with a better resolution than 4gen *I*_n_, and the 8,474 SNP analyses, especially in the Asian group (Figure 2).

Further analyses of the STRUCTURE results of the last set (4gen *pairwise *F_ST _SNP-set) via the coda package in the program R revealed full convergence of the Markov Chain of Monte Carlo (MCMC) in the STRUCTURE analyses in the Asian and Eurasian cluster, but not in the African and Native American clusters, suggesting an even more subtle subgrouping within these two groups (results not shown). A clusteredness analyses showed that the best fit for the total YCC dataset of 8,474 SNPs and of 47 ASMs (4gen *pairwise *F_ST_) was four major clusters (See additional file 2: rs-numbers of ascertained ASMs and additional information). As a final test, we analysed the 4gen *pairwise *F_ST _derived set of ASMs by means of Haploview in order to exclude strong linkage disequilibrium (LD) among the 47 SNPs. No significant LD could be detected (results not shown).

### ASM verification in independent samples

**Figure 3:**
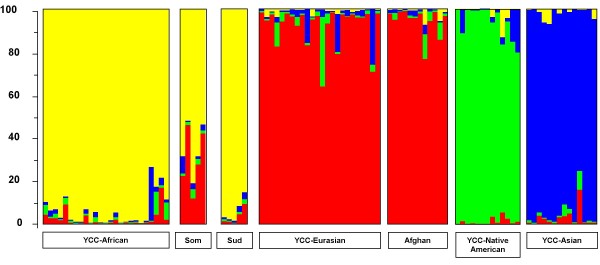
**STRUCTURE results after adding additional individuals to the YCC panel, based on 47 ASMs**. Genotypes of the 47 ASMs ascertained by the 4gen *pairwise *F_ST _approach from 22 individuals from Somalia, Afghanistan, and Sudan, were added to the genotypes of the original 74 YCC samples. After STRUCTURE analyses, the likelihood of the data was maximal at K = 4 ancestral populations (or clusters). Individuals are represented as vertical bars that are partitioned into segments corresponding to their membership of the clusters indicated by the four different colours. Each colour reflects the estimated relative contribution of one of the four clusters to that individual's genome and sum up to 100% (indicated at the Y-axis). Individuals were sorted according to their geographical origins (indicated below each group) after completion of the STRUCTURE analyses.

In order to verify that the ascertainment of the ASMs was not solely depending on the samples used for the initial marker ascertainment (the YCC panel), we added in a first verification step data from 22 individuals originating from Somalia (n = 5), Afghanistan (n = 12), and Sudan (n = 5) to the YCC data. This enlarged dataset was only analysed using STRUCTURE based on the 47 ASMs ascertained using the 4gen *pairwise *F_ST _approach. Samples from Somalia and Sudan grouped together with the African YCC-samples, whereas samples from Afghanistan grouped with the Eurasian YCC-samples, as one might expect on the basis of their geographical origin (see Figure 3).

**Figure 4:**
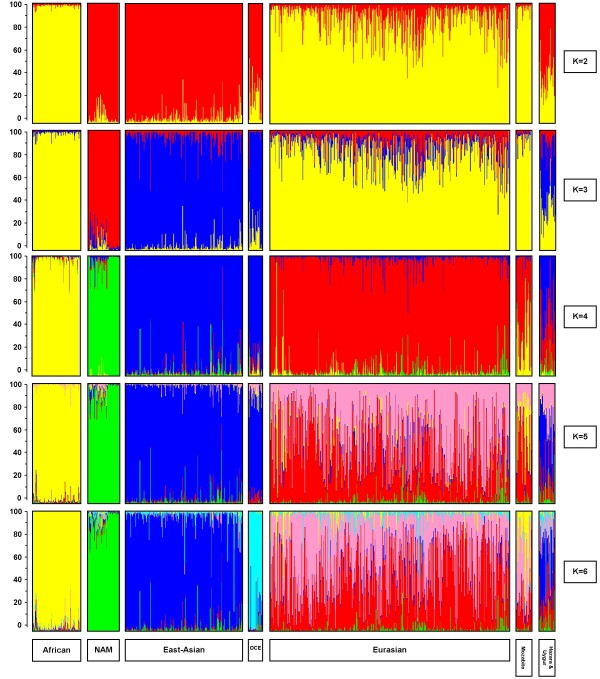
**STRUCTURE results with 47 ASMs in the HGDP**. The five horizontal groups show the results from STRUCTURE analyses for the H919 dataset genotyped for the 47 ASMs (ascertained with 4gen *pairwise *F_ST_) as proof of principle. We explored the estimated proportion of contribution of K ancestral populations (or clusters or subgroups) varying K from two to six. Individuals are represented as vertical bars that are partitioned into segments corresponding to their membership of the clusters indicated by two to six different colours. Each colour reflects the estimated relative contribution of one of the four subgroups to that individual's genome and sum up to 100% (indicated at the Y-axis). Individuals were sorted according to their geographical origins (indicated below each group) after completion of the STRUCTURE analyses.

In a second verification step, a series of STRUCTURE analyses were performed on the 47 ASMs resulting from the 4gen *pairwise *F_ST _approach genotyped in the H919 HGDP-CEPH dataset (Figure 4, see methods for a further explanation). With K = 2 STRUCTURE identified one cluster consisting of individuals from Asia (China, Siberia, Cambodia, Japan), Oceania, and the Native Americans, and another cluster consisting of individuals from Africa, Middle-East, Russia, Mozabite, and Europe. Defining three clusters (K = 3) resulted in the separation of the Asian and Oceanian individuals from Native Americans. Four clusters (K = 4) produced a similar result as for the YCC-data (compare Figure 2, 4gen *pairwise *F_ST_, with Figure 4 K = 4): all individuals from Africa clustered together, Native Americans formed a second cluster, individuals from Asia and Oceania were grouped together in a third cluster and individuals from the Middle-East, Russia, and Europe formed a fourth cluster. When increasing the number of clusters to five (K = 5), no distinct new cluster was formed, but individuals in the Eurasian cluster now appeared as mixed between two groups. When the number of clusters was further increased to K = 6, samples from Oceania formed a distinct additional cluster. Notably, in all STRUCTURE analyses, the Hazara (Pakistan) and Uygur (China) individuals showed a mixed genetic ancestry, reflecting both their Asian and Eurasian influences and the Mozabite individuals showed a mixed genetic ancestry from Africa and Eurasia. Further increasing the number of clusters did not result in the identification of more distinct genetic subgroups (not shown).

### ASM number limitations

**Figure 5:**
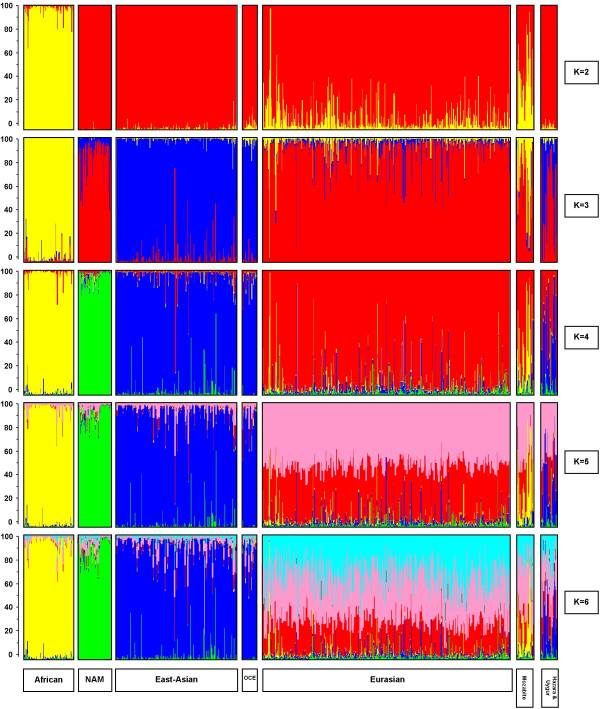
**STRUCTURE results with 34 ASMs in the HGDP**. The five horizontal groups show the results from STRUCTURE analyses for the H919 dataset genotyped for the reduced set of 34 ASMs (ascertained with 4gen *pairwise *F_ST_). We explored the estimated proportion of contribution of K ancestral populations (or clusters or subgroups) varying K from two to six. Individuals are represented as vertical bars that are partitioned into segments corresponding to their membership of the clusters indicated by two to six different colours. Each colour reflects the estimated relative contribution of one of the four subgroups to that individual's genome and sum up to 100% (indicated at the Y-axis). Individuals were sorted according to their geographical origins (indicated below each group) after completion of the STRUCTURE analyses.

Finally, we attempted to reduce the set of 47 ASMs resulting from the 4gen *pairwise *F_ST _approach genotyped in the H919 dataset by removing single SNPs in a one-by-one way followed by repeated STRUCTURE analyses. This resulted in a reduced set of 34 ASMs selected by the 4gen *pairwise *F_ST _procedure (See additional file 2: rs-numbers of ascertained ASMs and additional information). STRUCTURE analyses on this reduced 34 ASM dataset (Figure 5) revealed a somewhat different grouping than with the full set of 47 markers. In the K = 2 analysis Africans are now separated against the rest (whereas they were grouped with Eurasians against the rest with 47 ASMs). Subsequently, at K = 3 Eurasians appear grouped with Native Americans (whereas Native Americans appeared as separate group with 47 ASMs). At K = 4, the same grouping of Africans vs. Eurasians, vs. Asian/Oceanians vs. Native Americans was observed for 34 and 47 ASMs. At K = 5, the mixture of two clusters in the Eurasians was more prominent with the 34 than the 47 ASMs. At K = 6, the Oceanians could not be separated from the Asians with 34 ASMs as they were with 47 ASMs and a third component was detected in the Eurasian group not recognized with the 47 ASMs. Hazara/Uygur and Mozabite were also identified as admixed groups with the 34 markers.

## Discussion

The Mapping 10K array applied to the YCC samples proved to be a valuable tool for the identification of a set of ASMs with continental resolution. We found that STRUCTURE is a very useful program at various stages of the identification of ASMs. We demonstrated that the geographical distribution of samples is not necessarily the most optimal *a priori *stratification criterion. Barbujani and Belle [29] emphasize that if different sets of genetic data are analysed or the same dataset analysed with different methods, the same clusters should be found if the differences between populations and continents are large enough and consistent across loci. In our study, both different sample sets as well as different ascertainment methods were used and we consistently found four genetically distinct clusters of individuals corresponding to their different geographical origins being the best "fit" (Sub-Saharan Africans, Eurasians, East Asians (including Oceanians), and Native Americans). We also learned that ASM ascertainment by means of a single F_ST _(*classical*) estimate across all population samples or groups is not the most optimal strategy. *Classical *F_ST _values indicate the distinctive power of a SNP, however cannot identify directly for which population(s) this particular SNP could serve as a suitable ASM. For datasets with markedly different populations, *classical *F_ST _will most likely render SNPs that are specific for only the most distinct population(s), as we showed here for differences between Africans and non-Africans (additional file 1: F_ST _analyses of the five different sets of ASMs among the 74 YCC samples). Although STRUCTURE revealed that ASMs identified using *I*_n _(especially 4gen *I*_n_) resulted in a set of ASMs with a good clustering of the individuals in the YCC-panel into four major genetic groups, retrospective F_ST _analyses revealed that the *I*_n _based sets of 47 ASMs predominantly identified markers that distinguish African from non-African individuals. Only the use of 4gen *pairwise *F_ST _values provided ASMs with an optimal continental resolution of the YCC samples, and evenly distributed *pairwise *F_ST _values among all population pairs when compared with the other ascertainment criteria.

According to Gao and Starmer [30] ideal loci for distinguishing between populations, are those that have a fixed allele in one population and are absent in all other populations. In our method of screening (Mapping 10K) such SNPs could not be found due to the SNP selection procedures by Affymetrix, although other studies have been able to locate such SNPs using different screening methods. One example is the SNP analyses of skin pigmentation genes (e.g. MATP) by Norton *et al. *[31].

The apparent improvement of the genetic clustering of individuals using 47 SNPs (4gen *I*_n _and 4gen *pairwise *F_ST_) instead of 8,474 appears peculiar at first, but can readily be explained by interference of the SNPs with evenly distributed frequencies among populations. As such, the complete set of 8,474 SNPs reflects the most unbiased genetic make-up of all populations sampled, whereas the final set of 47 (or 34) ASMs only reflect the subtle genetic variation among populations maximized by means of extremely selected SNPs. STRUCTURE results (K = 4) were not altered after the addition of individuals originating from countries not present in the YCC panel (such as Somalia, Sudan, and Afghanistan). This can be interpreted as a first indication that the ascertained SNPs may indeed be reliable ASMs. Further analyses on a second much larger number of independent samples, the HGDP-CEPH (H919), proved independently the value of the selected ASMs for identifying the geographic origin of a DNA sample.

Our set of 47 ASMs identified using *pairwise *F_ST _was also able to detect mixed genetic ancestries. The individuals sampled in Algeria (Mozabite), appear to have a mixed ancestry between African and Eurasian descendants. The individuals from Hazara and Uygur exhibit both Eurasian and Asian genetic ancestries. This was also concluded by Rosenberg *et al.*[17,32] on the basis of a screening of samples from the HGDP-CEPH by means of 783 microsatellites and 210 insertion/deletion markers, as well as by Jakobssen *et al.*[33] and Li *et al.*[34] who analysed partially the same samples by means of > 500,000 and > 650,000 SNPs, respectively. As such, our results with the 47 ASMs following the 4gen *pairwise *F_ST _SNP ascertainment, are surprisingly similarly to the results based on the 40 most informative STRs identified by Rosenberg *et al. *[7]. Increasing the number of SNPs considerably does not necessarily improve the genetic clustering of the same HGDP-CEPH samples. For instance, Jakobssen et al [33] were only able to identify the Native Americans (NAM) as a distinct cluster at STRUCTURE analysis for K = 6, not at lower values of K. An even further increase in the number of SNPs (Li *et al. *[34]) resulted in STRUCTURE results at K = 4 that were similar to those obtained by us with 47 ASMs. Only upon increasing the values of K, Li *et al. *[34] were able to produce much more refined regional subclusterings. It is in this respect important to note that our final aim is to use a set of ASMs for forensic DNA research purposes. This imposes an important constraint on the amount of DNA available for an ancestry test, since rarely more than a few nanograms of input DNA is available, an amount of DNA that is not sufficient for aforementioned mass SNP genotyping platforms as used by us in this study (but also see e.g. [33,34]), but would be sufficient for a limited number of multiplex SNP assays enabling the genotyping of 40-50 SNPs. Reducing the 47 ASM-set ascertained based on *pairwise *F_ST _to 34 ASMs did not alter the STRUCTURE analyses with K = 4 but did show differences at other values of K.

Since the ASMs ascertained in this study were developed for distinguishing between the four major continental groups, individuals from other populations (e.g. Oceania (n = 2)) that were not (or under) represented in the YCC panel for marker ascertainment, were initially clustered within their most likely source population such as (East-)Asia in the case of Oceania. However, in the independent HGDP samples Oceania could be separated from East Asians at least with 47 ASMs and at K = 6. This illustrates that although the 47 ASMs were not explicitly selected to separate sub-groups within major continental regions, they do prove to be useful for this purpose at least in some cases.

Our *pairwise *F_ST _method for ascertaining ASMs ensures the absence of bias towards the African population, which usually shows the largest genetic differentiation compared to all non-African groups. This can easily be seen in the many studies analysing the HGDP-CEPH panel. In most analyses involving STRUCTURE or similar analytical tools the first split of the total group of individuals always could be related to a split between African versus non-African samples (e.g. [33]). At K = 2 our 34 ASMs, but not our set of 47 ASMs, showed a split between African and all non-African samples. A similar less prominent first split between African and non-African samples was also reported in other studies [17,34-36]. Despite these differences in clustering patterns for lower values of K, most studies report an identical major clustering pattern at K = 4 (Africa, Eurasia, East Asia (including Oceania) and Native America).

The ASMs ascertained in our study did not allow the differentiation among Eurasian individuals, i.e. individuals from Europe, Middle East as well as West and South Asia, which is similar to earlier findings based on a large number of STRs [17]. Finding ASMs with higher levels of specificity among Eurasian populations would improve the analyses considerably but obviously needs a different sampling design. Recently, two other largely overlapping studies were published [37,38] and demonstrated that the identification of the geographic sub-region of origin of an individual within Europe is possible within certain limits.

Other groups have also identified sets of ASMs that could differentiate the major geographical regions [39,40]. One example of such a different set of 34 SNPs is identified by Phillips *et al. *[36]. These SNPs do not overlap with our set of 34 and 47 ASMs, and were specifically sought in close proximity of genes that have been subjected to strong regional positive selection in the recent past [36]. Their set is able to distinguish between African vs. European, Middle Eastern - Central/South Asian vs. Oceanian, vs. East Asian vs. (Native) American groups also using the HGDP samples, similar to our findings, although at K = 4 it was not possible to distinguish Native American individuals from East Asian and Oceania. As such this is yet another indication that many other different sets of ASMs could be identified, depending on the *a priori *selection criteria.

## Conclusion

By means of a *pairwise *F_ST _ranking approach we identified a set of 47 SNPs that could serve as a panel of ASMs at a continental level. Multiplex genotyping (e.g. by means of SNaPshot^® ^genotyping) of such a restricted number of SNPs is feasible on the basis of only a few nanograms of input DNA. This not only enables genotyping these ASMs in DNA samples from population studies, but also makes it possible to analyse the limited amounts of DNA from forensic crime scene samples. Obviously, using such a set of ASMs and reporting the results in terms of prediction of ancestry in forensic casework is still in its infancy. The use of this set of ASMs (and others) among a much large number of samples from known globally distributed locations in order to verify and refine the possibility to predict the ancestry of an unknown sample is one of our next research priorities, as well as developing a simple statistical framework one can use to report ancestry probabilities.

## Methods

### DNA samples

We used a set of male DNA samples, released by the Y Chromosome Consortium (YCC) [25]. This set consists of 74 globally dispersed males and is subdivided in six geographical regions; (Central) Africa, South Africa, Asia (including Pakistan), Europe, Northern Asia (Russia and Siberia), and Native America. Each region consists of roughly similar numbers of individuals [25]. The samples in the YCC were either donated by volunteers who gave informed consent, donated by other research groups or purchased from the Coriell Institute [25]. For a first verification test 22 unrelated individuals from Somalia (n = 5), Afghanistan (n = 12), and Sudan (n = 5), selected from our immigration paternity casework samples, were genotyped for the set of ASMs finally ascertained. For the use of samples from these latter three populations, permission was granted by the responsible Dutch immigration authorities under the condition that the samples would remain fully anonymous whilst retaining the country of birth of each individual. Subsequently, proof of principle was obtained by testing the CEPH Human Genome Diversity Panel (HGDP-CEPH) [28]. The complete HGDP-CEPH consists of 1,064 individuals from 51 globally distinct populations. The use of HGDP-CEPH samples is fully explained by the initiators of the sample repository [28]. All selected ASMs were also genotyped in the full HGDP-CEPH. Before statistical analyses, all sample duplicates, samples with labelling errors, and all closely related individuals where removed, resulting in a set of 952 HGDP-CEPH individuals (H952) [7,17,41,42]. During the course of our analyses we found out that H952 contained 33 YCC panel samples which were subsequently removed resulting in a final non-overlapping HGDP-CEPH panel of 919 unrelated individuals (H919).

### SNP genotyping for ASM ascertainment

Throughout this manuscript we use the word "ascertainment", instead of other often used words as "identified" or "selected" to indicate the full process of ASM marker discovery. This process, due to the nature of the markers used in this study, did not use objective and strict criteria or thresholds of, e.g. allele frequencies.

All 74 individuals from the YCC panel and the additional 22 individuals from Somalia, Afghanistan, and Sudan were genotyped for the 11,555 SNPs included in the Mapping 10K array according to the Affymetrix protocol for the GeneChip^® ^Mapping Human 10K Array (GeneChip^® ^Mapping Assay Manual). These SNPs have a median physical distance of 105 kb, an average distance 210 kb, and an average heterozygosity of 0.37 in the Affymetrix test panel. After hybridisation, the arrays were washed, stained, scanned and analysed with the software supplied by Affymetrix (Affymetrix Inc., Santa Clara, CA) [43].

The Mapping 10K arrays produced a dataset of 878,180 SNPs (11,555 per individual), however not every SNP tested resulted in a reliable genotype call. On average, the arrays genotyped 92 percent of the SNPs per individual (default settings in Affymetrix software), which effectively represents approximately 10,635 SNPs. From this dataset we selected only those SNPs for which 90% or more of the 74 YCC individuals had a valid genotype. This resulted in 8,650 SNPs per individual. Subsequently, we excluded all SNPs on the X-chromosome (n = 176), leaving a final dataset of 8,474 SNPs. The 8,474 SNP-set was used for further analyses.

### Population structure analyses

We initially explored the dataset of 8,474 SNPs among the 74 YCC individuals by means of the program STRUCTURE version 2.1 and 2.2 [see reference [20] and the STRUCTURE manual for detailed information]. STRUCTURE can be used to identify genetic clusters among a set of individuals on the basis of multilocus genotypes. Assuming admixture among populations, correlated allele frequencies, and no prior population information we used STRUCTURE to explore different number of possible clusters (K) from 1 to 8. For each K, a maximum of 20 runs were performed with a burnin length 20.000 iterations followed by 10.000 MCMC iterations We tested for convergence using the coda Package in the program R (available on ). Clusteredness was tested following equation 3 from Rosenberg *et al. *[32]. STRUCTURE was used (i) for the ascertainment of the most optimal number of distinct clusters, (ii) to subsequently test the performance of different sets of selected ASMs, and (iii) for the proof of principle analyses. The output of STRUCTURE is in the form of a structural map showing the relative estimated proportion of contribution of K ancestral populations (or clusters or subgroups) for each individual. In Figure 1 (showing the result of the best-fit- STRUCTURE analyses of 8,474 SNPs among the 74 YCC individuals) each vertical bar represents a single individual and each colour a distinct ancestral population. Upon running STRUCTURE, the best "fit" (i.e. the most optimal number of K) was four, hence the four different colours in this figure. Individuals are analysed without any prior population information, but are sorted once STRUCTURE is completed by their sampling population. The STRUCTURE output graphs in Figures 1, 2, 3, 4, and 5, were produced using Microsoft Excel and Microsoft PowerPoint based on the raw STRUCTURE output tables.

### ASM ascertainment

Our dataset comprised of six geographically defined groups (6geo). Instead of relying on this *a priori *sample stratification, we performed STRUCTURE analyses to obtain a more unbiased estimate on the number of (genetically) distinct subgroups. Structure analyses on the full dataset (8,474 SNPs) revealed a best fit at four genetically distinct subgroups (4gen). These four genetic subgroups reflect the four different continental origins of all samples.

In order to identify possible ASMs we initially used two estimators: F_ST _(among population F-statistics) [26] and *I*_n _(Informativeness of assignment or ancestry) [7]. Classical F_ST _is a measure of population differentiation based on polymorphic genetic data, such as single nucleotide polymorphisms. It compares the genetic variability between any number of populations. When used among more than two *a priori *defined populations it provides an overall estimate and does not indicate which specific population or populations is genetically more distinct. The *I*_n _statistic computes the potential of assigning an allele to one of the defined (genetically or *a priori*) clusters considering all the clusters in the study (e.g. six geographical sampling areas or four major continental groups) [7].

In a combined dataset one or more subgroups or clusters could be more genetically distinct from the others. The *I*_n _statistic is proportional to the total amount of differentiation between populations and is expected to be less influenced by more genetically distinct subgroups. However, in classical F_ST _calculations this could result in the ascertainment of ASMs specific for a single most genetically distinct cluster. To avoid this, we used a third strategy in which F_ST _values (*pairwise *F_ST_) were estimated by only comparing two clusters (genetically defined or *a priori*) at a time instead of two or more in classical F_ST _analyses. Pairwise F_ST _values were computed for all possible *pairwise *comparisons among the dataset. High *pairwise *F_ST _values were sought by comparing the groups for all possible *pairwise *comparisons among the dataset, guided by the classical F_ST _values. *Pairwise *F_ST _were estimated following a geographically defined distribution of the dataset (6geo) as well as a genetically distinct distribution (4gen). For the classical F_ST _values the 97.5 percentile upper boundary was calculated and all *pairwise *F_ST _values higher than or equal to this value were given a value of one. SNPs with a value of one for all *pairwise *comparisons or certain specific ones (for example Asia versus all other continental groups except for instance Africa) were considered suitable for ascertaining as ASMs.

For the ascertainment of ASMs, all SNPs were simply ranked according to the highest values of F_ST _and *I*_n_. Subsequently, the top 50 SNPs with highest classical F_ST _and *I*_n _values were selected from the six geographically *a priori *defined groups (6geo) distribution of our dataset as well as the four genetic posteriori defined clusters (4gen) distribution (the latter identified through STRUCTURE analyses). The *pairwise *F_ST _ascertainment of ASMs was restricted to the dataset with the genetic distribution (4gen) of individuals, since the geographic distribution (6geo) yielded too few group-sensitive SNPs due to erroneous *a priori *geographical assignments of samples. Initially, similar amounts of group-specific SNPs (ASMs) were obtained per major continental group. However, some SNPs could not be reproduced via the TaqMan assay and were omitted from the ASM-set, leaving a set of 47 ASMs. As the omission of these SNPs did not alter the STRUCTURE results, no new SNPs were selected as ASM and from the other SNP-sets (following from classical F_ST _and *I*_n _computations) the last three SNPs from the top 50 SNPs were removed, leaving the top 47 loci for further analyses. To summarize, by means of 5 different ascertainment procedures we designed 5 different sets of 47 SNPs that could potentially serve as ASMs.

By means of Haploview [44] we tested the selected SNPs for possible linkage disequilibrium (LD), and no significant LD could be detected.

### SNP genotyping for ASM verification

The selected ASMs were genotyped in the HGDP-CEPH via the TaqMan^® ^Technology. Of each sample three nanograms of DNA were dried in open air in 384-well clear optical reaction plates (Applied Biosystems, Foster City, CA, USA). Two μl of amplification mix was added containing 1 μl Absolute QPCR rox mix (ABgene, Epsom, UK), and either 0.1 μl 20× Custom TaqMan^® ^SNP Genotyping Assays (Applied Biosystems) or 0.05 μl 40× TaqMan^® ^SNP Genotyping Assays (Applied Biosystems). Amplification (15' 95°C, 40 cycles 15" 95°C and 1' 60°C) was performed in a GeneAmp 9700 PCR cycler (Applied Biosystems) followed by a subsequent end-point reading on an Applied Biosystems 7900 HT Fast Real-Time PCR System according to the manual of the manufacturer. Assay numbers or primer and probe sequences in case of assay on design are provided as supplement material (see addition file 3: TaqMan Assay).

## Authors' contributions

PK carried out part of the labwork, was involved in data analyses, conducted SNP ascertainment analyses, performed all other statistics and wrote the manuscript. KvD carried out the HGDP TaqMan analyses. ADK provided comments on the manuscript. JTdD provided facilities for array-handling in the Mapping 10K assay. MK designed and coordinated the HGDP analyses, and provided revisions of the manuscript. PdK designed and supervised the study and made final improvements of this manuscript. All authors read and approved the final manuscript.

## Supplementary Material

Additional file 1 **F_ST _analyses of the five different sets of ASMs among the 74 YCC samples**. This file lists the results of a retrospective F_ST _analysis of all 74 YCC samples grouped into the four genetically defined subgroups or six geographically defined subgroups with each of the five different sets of 47 ASMs. Click here for file

Additional file 2 **rs-numbers of ascertained ASMs and additional information**. This file lists the rs-numbers and additional information of two sets of ASMs identified in our study. The 47 ASM set represents the final set of 47 ASMs identified by the pairwise F_ST _4gen procedure. The 34 ASM set represents a further reduction of this 47ASM set. Click here for file

Additional file 3 **TaqMan assay**. Primer sequences and additional information for the SNPs genotyping using the TaqMan assay. Click here for file
